# The femur too short? 1373 fetuses with short femur during second-trimester screening

**DOI:** 10.1007/s00404-021-06394-z

**Published:** 2022-01-11

**Authors:** Ulrike Friebe-Hoffmann, Larissa Dobravsky, Thomas W. P. Friedl, Wolfgang Janni, Alexander J. Knippel, Hans J. Siegmann, Peter Kozlowski

**Affiliations:** 1 Ulm, Germany; 2 Prenatal Medicine & Genetics, praenatal.de, Düsseldorf, Germany

**Keywords:** Short femur, Prenatal, SGA, Malformation, Aberration

## Abstract

**Purpose:**

A short fetal femur in prenatal diagnosis might be an indicator for intrauterine growth retardation (IUGR), a genetically determined small child (SGA) with or without associated fetal malformations and/or an adverse fetal outcome.

**Methods:**

1373 singleton pregnancies with a femoral length < 5th percentile detected between 1999 and 2015 during second-trimester screening in a tertiary prenatal diagnostic center were subjected to a descriptive retrospective analysis with regard to fetal characteristics as well as pregnancy outcome.

**Results:**

685 (49.9%) fetuses presented an isolated short femur, while 688 (50.1%) showed additional abnormalities. 293 (42.6%) of those were SGA babies without any malformation, while 395 (57.4%) had one or more severe anomaly of the following organ systems: 157 (11.5%) cardiovascular, 101 (7.4%) musculoskeletal, 82 (6.0%) urogenital, 72 (5.2%) cerebrocephalic, 50 (3.6%) gastrointestinal, and 5 (0.4%) thoracic. 75 (5.5%) of the fetuses showed chromosomal aberrations of which Trisomy 13, 18 and 21 were found in 2, 13 and 27 of the cases, respectively. Fetuses with associated malformations had a significantly lower live birth rate than those without (64.2% vs. 98.1%, *p* < 0.001); in addition, a higher rate of preterm births 36.6% vs. 11.3%, *p* < 0.001) and SGA babies (51.4% vs. 30.4%, *p* < 0.001) were observed in the first collective.

**Conclusion:**

Diagnosis of a short fetal femur should lead to an extended organ screening; in the case of associated abnormalities, additional genetic testing has to be offered, as well as intensified pregnancy monitoring in pregnancies at risk for IUGR and/or preterm birth.

## Introduction

While during the first trimester of pregnancy, fetal crown-rump length and biparietal diameter are used to measure and confirm gestational age, a combination of the fetal biparietal diameter, the head circumference, the abdomen diameters and its circumference as well as the femur length of the baby is used during second and third trimester for prenatal growth estimation and weight assessment. Hereby, the measured length of the femur is elementary [1].

In 1994, Snijders and Nicolaides first established percentiles to define normal ranges for biometric parameters. A short fetal femur was defined as a femur length below the 5th percentile [2].

Up to now, the discriminatory power of a prenatally diagnosed “short femur” has been discussed heterogeneously as it may indicate early growth restriction [3–5], be a soft marker for trisomy 21 [6–11], suggest skeletal dysplasia [4, 5, 12] and also show as a norm variant, especially in the context of different ethnicity [13, 14]. The aim of this study was to determine the extent to which the sonographic diagnosis of a “short femur” is associated with poorer fetal outcome on the basis of an exceptionally large, representative data pool. In the presented study, we decided to use current growth curves [15] to assess fetal femur length, and particularly paid attention to additional fetal malformations, chromosomal defects and birth-related risks such as small for gestational age babies (SGA), preterm birth (PTB) and low birth weight (LBW) to get a fundament for adequately counseling parents with fetuses affected.

## Materials and methods

Our outpatient practice is highly specialized for prenatal diagnostics with about 15.000 patients a year. A search of the database between 1999 and 2015 was performed to retrieve all cases with a short femur < 5th percentile during second-trimester screening (18 + 0–21 + 6 weeks of gestation) according to the calculation of Verburg et al. [15]. A retrospective analysis was then performed on all singleton pregnancies diagnosed with a short fetal femur.

Gestational age was calculated by the first day of the last menstrual cycle and confirmed by crown-rump length during early ultrasound examination before 12 + 0 weeks of pregnancy. If the difference between clinical and ultrasound dates was more than 7 days, gestational age was corrected according to the earliest ultrasound findings.

Besides biometric calculations, all fetuses with a short femur underwent detailed anatomic scanning, and karyotyping was offered to all women with additional fetal abnormalities or was performed at patient´s request. After the exclusion of incomplete or implausible data sets as well as compilation of repetitive examinations of a fetus, 1373 cases were subjected to a more detailed analysis. The assessments were performed by five highly specialized (DEGUM II-III) sonographers with the most modern ultrasound systems of the time (list at the authors). Data on prenatal findings as well as perinatal outcome were collected.

Statistical analysis was performed using SPSS Statistics version 21 software (IBM, Armonk, NY, USA). Data on categorical variables were summarized using absolute and relative frequencies, and associations between categorical variables were analyzed with the Chi-squared test or Fisher’s exact test (in case of expected frequencies of less than 5 in 2 × 2 contingency tables). As the distribution of all quantitative (metric) variables included in this study differed significantly from normal (Shapiro–Wilk test, all *p* < 0.001), these variables were described using median and interquartile ranges. Statistical comparisons between groups with regard to metric variables were performed by the non-parametric Mann–Whitney *U* test. All statistical tests reported here were two-tailed, and a *p* value ≤ 0.05 was considered statistically significant. The study has been granted an exemption from requiring ethics approval by the local ethics committee of Ulm University.

## Results

During the period of 1999 and 2015, out of 138.238 singleton pregnancies scanned during second trimester, a total of 1373 cases presented with a short femur < 5th percentile according to Verburg et al. [15] and were eligible for further analysis by having complete information on structural and chromosomal abnormalities.

Median age of women was 31 years (range 15–49), 97% of women of whom data of ethnicity was recorded (*n* = 202) were Caucasian.

Placental structure and amniotic fluid were inconspicuous in more than 90% of cases (93.4% and 97.1%, respectively).

Among the 1373 fetuses with a short femur, 1213 (88.3%) live-born children were documented, while 28 (2%) perinatal deaths, 64 (4.7%) miscarriages or intrauterine demises and 68 (5%) voluntary terminations, of whom nearly 90% were performed before 24th week of gestation, were registered.

Birth weight was documented for 1241 (90.4%) of the live births. Median birth weight was 3050 g (200–5090 g), while 23.0% (*n* = 286) had a birth weight below 2500 g.

The proportion of SGA children of the 1213 live births was 34.3% (*n* = 416). The total percentage of preterm infants was 15.5% (*n* = 188), of which 30% were very preterm infants born before 32 + 0 weeks of gestational age.

A differentiated analysis of the association between preterm birth and SGA revealed that SGA fetuses were born significantly more were born more often as premature babies than non-SGA fetuses (*p* < 0.001).

While half of the fetuses (49.9%, *n* = 685) presented with an isolated short femur, the other half (50.1%, *n* = 688) showed additional abnormalities: 293 (42.6%) of those were SGA babies, while 395 (57.4%) had one (*n* = 211) or more severe malformation.

**Fig. 1:**
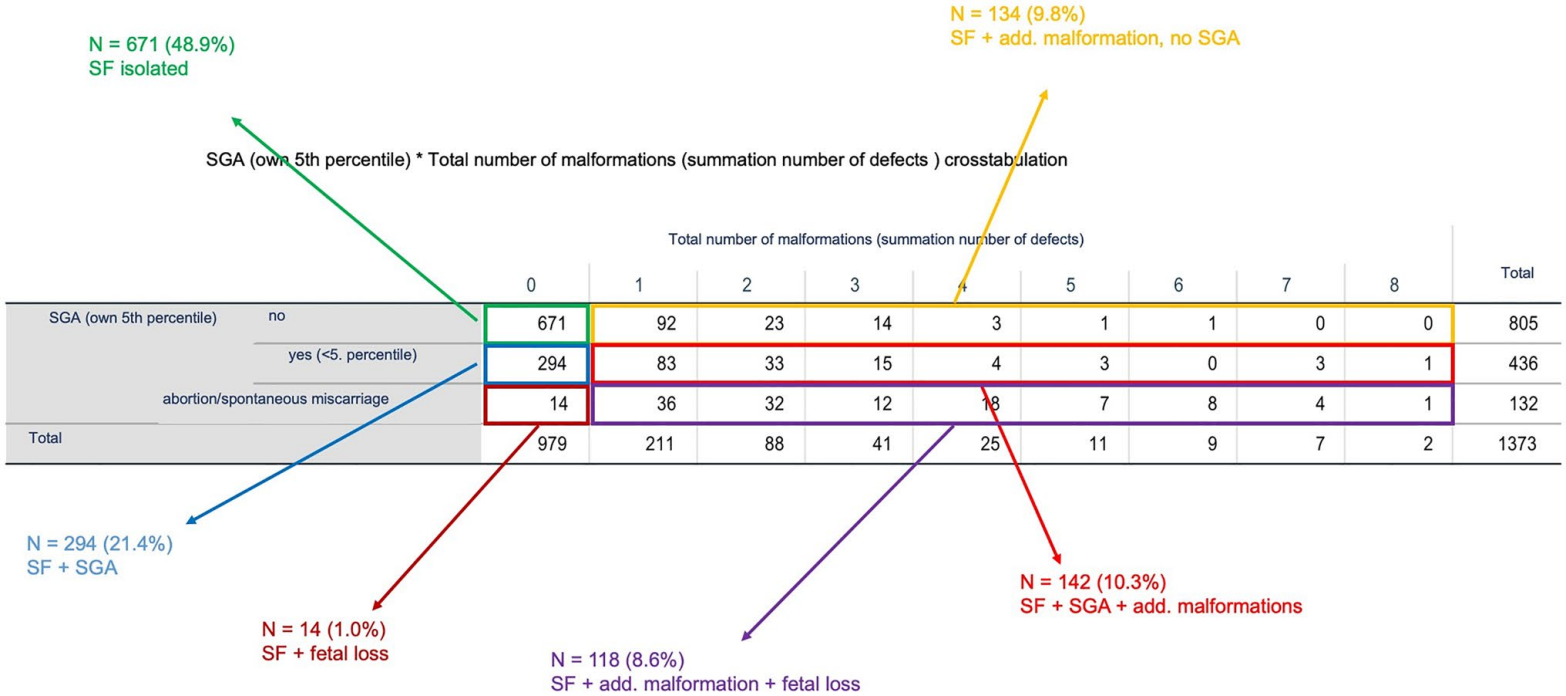
Correlation of short femur with/without malformation and SGA

The following organ systems were: 157 (11.5%) cardiovascular, 101 (7.4%) musculoskeletal, 82 (6.0%) urogenital, 72 (5.2%) cerebrocephalic, 50 (3.6%) gastrointestinal, and 5 (0.4%) thoracic (shown in Fig. 1, Table 1).

**Table 1:** Structural malformations by the organ systems involved in 1373 fetuses with a short biometric femur (688 fetuses with at least one structural malformation)

Organ system	Total (*N* = 688)
Cardiovascular malformations	**n = 194**
White spot	53
Atrial septal defect	4
Ventricular septal defect	31
Atrioventricular septal defect	14
Tricuspid valve abnormality	4
Aortic valve abnormality	5
Pulmonary valve abnormality	2
Tetralogy of Fallot	9
Pulmonary stenosis	1
Coarctation of the aorta	9
Right aortic arch	2
Transposition of great arteries	5
Double-outlet right ventricle	3
Double-outlet left ventricle	1
Hypoplastic right heart syndrome	1
Hypoplastic left heart syndrome	1
Malposition of the heart	3
Cardiomegaly/insufficiency	4
ARSA	2
Miscellaneous (not specified)	40
Musculoskeletal malformations	**n = 118**
Bone dysplasia/aplasia	34
Other malformations of the extremities	68
Malformation of the feet	15
Malformation of the hands	19
Nasal bone hypoplasia/aplasia	2
Abnormal facial profile/micrognathia	13
Skeletal dysplasia/Arthrogryposis	11
Cleft lip/palate	9
Diaphragmatic hernia	2
Gastrointestinal malformations	**n = 55**
Hyperechogenic bowl	20
Gastrointestinal atresia	10
Omphalocele	6
Gastroschisis	5
Ascites	4
Tumor/cyst of the liver	3
Hepatomegaly	2
Miscellaneous	5
Cerebro-cephalic malformations	**n = 88**
Plexus cyst	26
Hydrocephalus/ventriculomegaly	23
Dandy–Walker-malformation	9
Abnormal head shape	7
Cerebellar hypo-/aplasia	5
Spina bifida aperta	4
Corpus callosum agenesis/dysgenesis	5
Microcephalus	2
Holoprosencephaly	1
Anencephaly	1
Urogenital malformations	**n = 92**
Single umbilical artery	29
Hydronephrosis	20
Renal dysplasia/agenesis unilateral	8
Renal dysplasia/agenesis bilateral	7
Renal duplication/ectopia	9
Hypospadia	4
LUTO	2
Thoracic malformations	**n = 6**
Hydrothorax	2
Pulmonary dysplasia	2
CHAOS-syndrome	2

In our data set, more than 86% of diagnosis were have been made prenatally, just 13.46% (102) malformations were encoded postnatally.

75 (5.5%) of the fetuses showed chromosomal aberrations. In 14 cases, structural aberrations were found, while 61 fetuses presented with numeric aberrations of which Trisomy 21, 18, 13, Monosomy X (45, X0) or Triploidy were found in 27, 13, 2, 10 and 6 cases, respectively. The majority of these fetuses (81.3%, *n* = 61) carried additional malformations besides a short femur and their mothers were slightly older (33 years vs. 31 years) than the average age of the cohort.

Table 2 shows the different types of chromosomal disorders found in the cohort.

**Table 2:** Type of chromosomal disorder found in the cohort listed by numbers

Type of chromosomal disorder	Total (*N* = 75)
Trisomy 21	27
Trisomy 18	13
Monosomy X	10
Triploidy	6
Inversion	4
Other autosomal numeric chromosome disorder	3
Gonosomal structural disorder	3
Autosomal unbalanced structural chromosomal disorder	2
Trisomy 13	2
Ring chromosome	2
Translocation	1
Autosomal balanced structural chromosomal disorder	1
Duplication	1

**Fig. 2:**
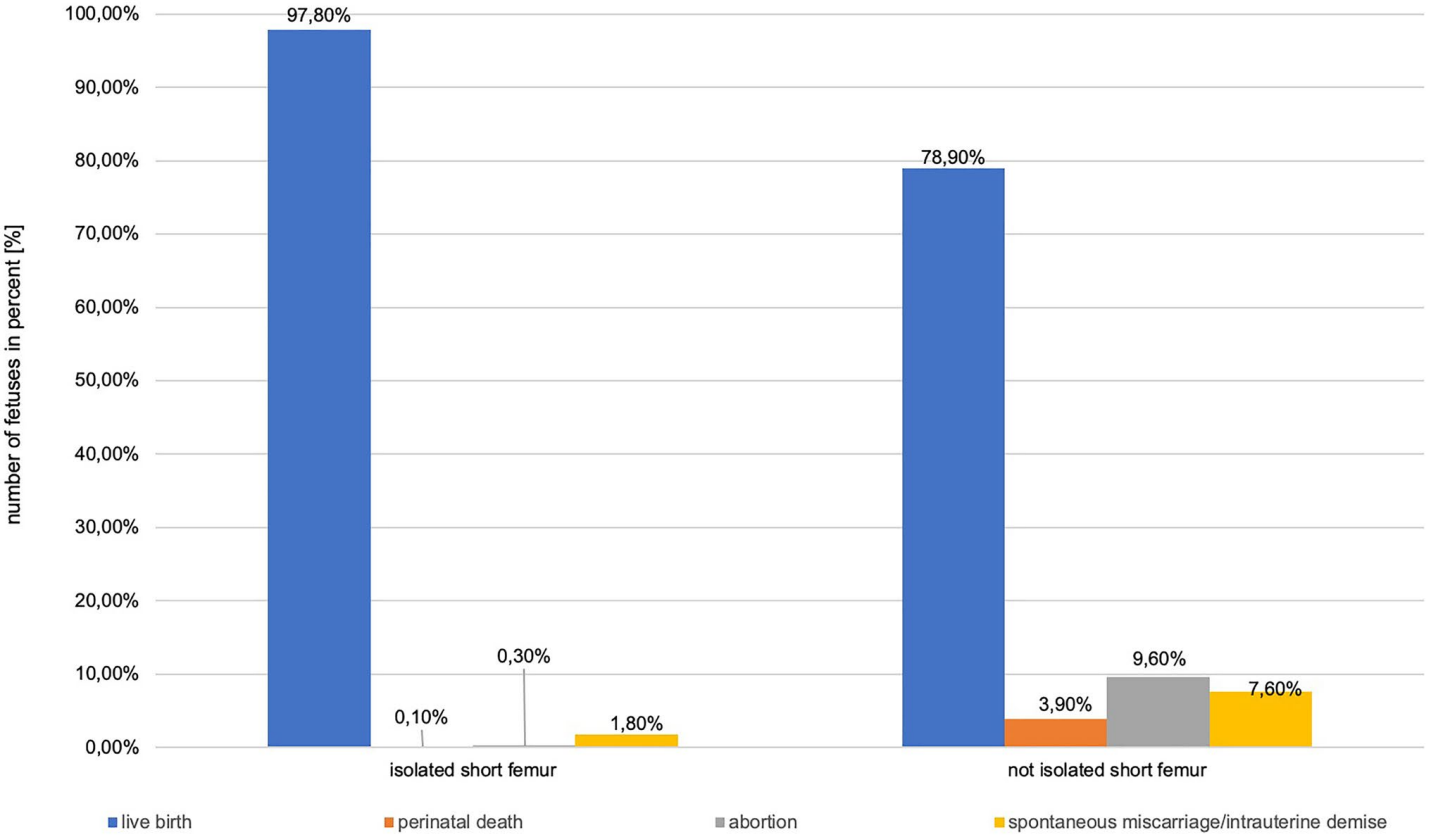
Outcome of fetuses with isolated short femur vs. not isolated short femur in %

Children with isolated short femur differed significantly in terms of outcome from children with short femur and additional abnormalities (SGA and/or other malformations); children with an isolated short femur had a higher live birth rate (97.8% vs. 78.9%) and a lower rate of perinatal death (0.1% vs. 3.9%), abortions (0.3% vs. 9.6%) or spontaneous miscarriages/intrauterine demises (1.8% vs. 7.6%) (shown in Fig. 2).

**Fig. 3:**
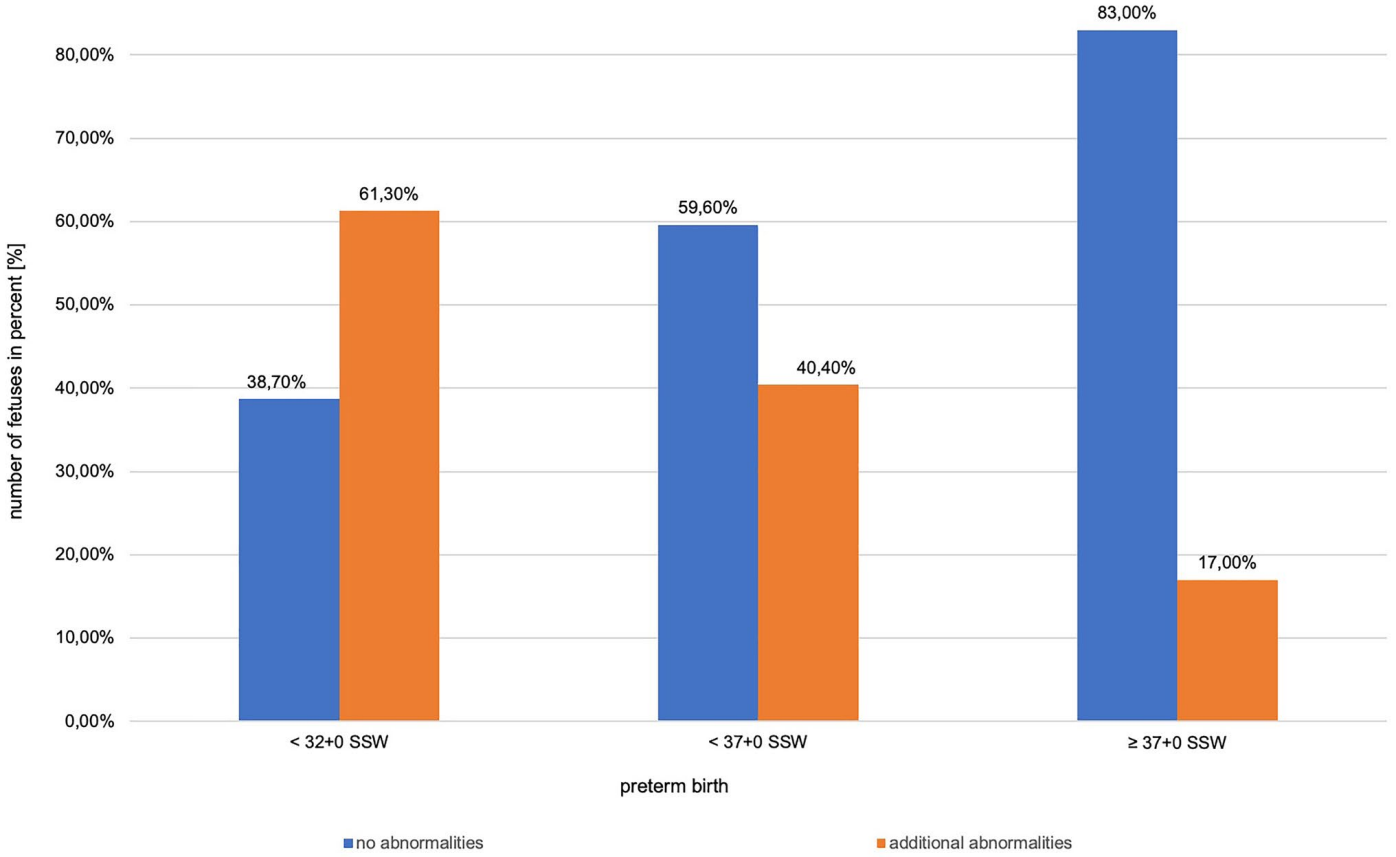
Number of fetuses in % without or with malformations delivered preterm

In addition, live-born children with malformations were significantly more often preterm than children without additional abnormalities (*p* > 0.001) (shown in Fig. 3).

## Discussion

The development of prenatal medicine is progressing very rapidly. Particularly in the field of genetic diagnostics, possibilities have grown immensely in recent years.

Fetal ultrasound is the basis ultrasound is the basis of every prenatal diagnosis, and the more precise the examination, the more precise this leads to a clear diagnosis and thus forms the fundament of optimized counseling for expectant parents.

Sonographic assessment of the femur length is one of the routine steps towards biometric measurement and, in the age of high-resolution ultrasound equipment, easy to perform. A short femur is defined by a femoral length below the 5th percentile of the standard curves of defined biometric measurement parameters, including normal healthy children with short stature as a standard variant. Furthermore, a short femur is considered to be a so-called soft marker, which, in combination with other soft markers, increases the risk of trisomies, especially trisomy 21. An association with additional fetal malformations, chromosomal defects, placental insufficiency or other birth-related risks such as preterm birth and low weight infants has been postulated [16]

1373 singleton pregnancies with a femoral length < 5th percentile [15] detected between 1999 and 2015 during second-trimester screening in a tertiary prenatal diagnostic center were subjected to a descriptive retrospective analysis with regard to fetal characteristics as well as pregnancy outcome.

One might argue that the number of fetuses eligible for analysis did not exceed 1% of the total collective seen in our center during the period of data collection; nonetheless to the best of our knowledge, no bigger data pool of fetuses with a short femur has ever been examined before. The biggest data set was published by Mathiesen et al. in 2014 [17]. The definition of a short femur varies in recent literature and only few studies distinguish between an isolated short femur and biometric short femurs in combination with further malformations or in the context of a syndrome, thus making it challenging to compare these works with each other.

Ethnicity was not documented in clinical practice during the first ten years of data collection. The influence of various factors, such as maternal and paternal height, BMI or birth weight, as well as ethnicity on medical conditions, only emerged at the beginning of the 2000s. In the present study, however, due to the local background as well as the survey period, it can be assumed that the vast majority of the Düsseldorf collective that was studied were women of Caucasian origin which is strengthened by the proportion of 97.0% Caucasian women of the 202 registered ethnicities.

Skeletal dysplasia has been found in about 4–15% of collectives with short femur measurements [4, 5, 18, 19]. While our data included 7.4% musculoskeletal abnormalities, just 0.8% of the cohort presented with skeletal dysplasia. This might be due to the fact, that in former times karyotyping as the only genetic diagnostic being remunerated by our health system, did not lead to early detection of determined skeletal dysplasias, while nowadays, in the time of microarrays & co. the detection of it might rather lead to early pregnancy termination.

Todros et al. [5] and Papageorghiou et al. [4] found 36–46.5% structural abnormalities among short femur fetuses, while in our study, half of the cases presented with additional malformations. In our dataset, a predominance of anomalies of the cardiovascular system (14%) was found, while other authors just identified 1.8% cardiovascular malformations among 22% structural abnormalities in their collectives of short femur fetuses [5, 19]. However, works roughly coincide showing 2.7–4.6% urogenital, 1.8–3.9% cerebro/cephalic, 0.9–2.3% gastrointestinal, 2.7% thoracic and 0.8–5.8% other malformations, much less than we found in the current study. Accordingly, short femur fetuses in all studies showed urogenital malformations most frequently, followed by cardiovascular and musculoskeletal abnormalities as mentioned above, followed by cerebro/cephalic abnormalities. Deviations in percentages can be explained by different study designs, different examination times during pregnancy and much smaller case numbers in the above-mentioned works in contrast to our comprehensively examined large data pool.

Unfortunately, the only large cohort of short femur fetuses examined by Mathiesen et al., exclusively considered additional genetic abnormalities in these cases disregarding structural malformations [17].

Soft markers as, e.g., an intracardiac echogenic focus, not counting as a malformation per se, were not disregarded in the present work, because of their correlation to chromosomal abnormalities, especially to Trisomy 21. Nevertheless, with 10.3%, there still remains a high incidence of malformations within this organ system, if excluded from the subcategory of cardiovascular abnormalities.

Most malformations occurred, as far as detected, without detectable genetic changes, but most genetically altered fetuses had malformations.

From today’s point of view, the number of genetic aberrations seems to be small, due to the fact, that at the time when data collection has started, possibilities to collect information about monogenic diseases, submicroscopic inversions or microdeletions were not available in routine diagnostics. We think, that a prospective study conducted nowadays would lead to much more information about the underlying genetic conditions behind short femur kids, as could be shown in a small study by Liu et al. in 2019 [20].

Previous studies have shown a correlation between a short fetal femur and an increased incidence of small for gestational age and/or IUGR fetuses [21, 22]. In the present study group, 31.8% of women gave birth to an SGA child, which is consistent with the documented prevalence of 14–43% in other surveillances [5, 16, 17, 23–27].

Numerous research works have described an association of short fetal femur length and low birth weight neonates [5, 23]. We found 23.0% LBW fetuses in our cohort, accordingly others found frequencies of 17.5%-23.9% LBW fetuses among short femur children, compared to 7.1%-8.6% LBW fetuses with normal femur biometry [24, 26–28].

With regard to preterm birth, the literature shows contradictory results: while some studies could not find any significant differences between an isolated short femur group and comparative collectives [23, 24], larger cohort studies report a significant increase in preterm birth rates up to 20% in children with short thigh length [25–28].

The results of the present study showed an overall increased preterm birth rate of 15.4% (*n* = 211). Within this group, 5.3% children with isolated shortened femur were born preterm vs. 25.4% of fetuses with additional abnormalities and/or SGA (*p* < 0.001).

As a descriptive retrospective study, the presented analysis has its limits: the value of our data reflects that of a middle European population being recruited by a single center and reflecting the referral base of the institution. Aneuploidy rate and outcome variables are those of fetuses seen for organ screening in the second trimester. Due to the fact, that underlying chromosomal anomalies as, e.g., trisomy 13 or 18 tend to be limited at earlier gestational ages, the overall rate of associated anomalies and chromosomal disorders in short femur cases might be even higher.

Due to the non-centered medical care in a large metropolitan area with a high number of birth centers, it was nearly impossible over the time period of 16 years to obtain a standardized follow-up of the neonates in retrospective, certainly a shortcoming of the study, as so often in retrospective analyses.

A major strength of the study is the large sample size with an overall number of 1373 fetuses with a short femur including 685 cases with an isolated femur < 5th percentile. To the best of our knowledge, so far, the presented work is the largest single study to evaluate short femur in prenatal ultrasound examination and its correlation to fetal outcome.

## Conclusion

In summary, within 1373 fetuses analyzed, a femur below the 5th percentile in 49.9% of cases was an isolated symptom.

Besides 14 cases of miscarriage/abortion, the later were born as otherwise healthy newborns. Though intense sonographic surveillance is not needed in the later, it will most likely be performed by suspicion of evolving complications, uncertainty of a sonographer or need for safety by the mother-to-be.

28.7% of the fetuses showed additional abnormalities, including 23.2% structural malformations and 5.5% chromosomal abnormalities. The present study emphasizes that a qualified ultrasound during pregnancy is not only able to perform fetal weight estimation but also might detect, if irregular, possible associated fetal problems.

In any case of additional structural abnormality, genetic testing should be offered. If a short femur is found to be part of a small for gestational age baby, an intrauterine growth retardation or a suspected late growth retardation, standard pregnancy care should be intensified by additional intervallic biometry and Doppler assessment on a 2 weeks basis.

In the absence of studies combining prenatal investigations with prospectively planned postnatal follow-up, the real impact of a short fetal femur on perinatal outcome and especially long-term development of affected children remains unclear. Further data collection and prospective studies are warranted to determine best prenatal care of unborn with short femur.
